# An experimental study of the Online Information Paradox: Does en-route information improve road network performance?

**DOI:** 10.1371/journal.pone.0184191

**Published:** 2017-09-13

**Authors:** Kasun P. Wijayaratna, Vinayak V. Dixit, Laurent Denant-Boemont, S. Travis Waller

**Affiliations:** 1 Research Centre for Integrated Transport Innovation (rCITI), School of Civil and Environmental Engineering, University of New South Wales, Sydney, Australia; 2 University of Rennes1, Department of Economics, Center for Research in Economics and Management (CREM-CNRS), Rennes, France; Beihang University, CHINA

## Abstract

This study investigates the empirical presence of a theoretical transportation paradox, defined as the “Online Information Paradox” (OIP). The paradox suggests that, for certain road networks, the provision of online information deteriorate travel conditions for all users of that network relative to the situation where no online information is provided to users. The analytical presence of the paradox was derived for a specific network structure by using two equilibrium models, the first being the Expected User Equilibrium (EUE) solution (no information scenario) and the other being the User Equilibrium with Recourse (UER) solution (with information scenario). An incentivised computerised route choice game was designed using the concepts of experimental economics and administered in a controlled laboratory environment to investigate the physical presence of the paradox. Aggregate statistics of path flows and Total System Travel Costs (TSTC) were used to compare the experimental results with the theoretical findings. A total of 12 groups of 12 participants completed the experiment and the OIP and the occurrence of the OIP being significant was observed in 11 of the 12 cases. Though information increased travel costs for users on average, it reduced the volatility of travel costs experienced in the no information scenario indicating that information can achieve a more reliable system. Further replications of similar experiments and more importantly field based identification of the phenomena will force transport professionals to be aware of the emergence of the paradox. In addition, studies such as this emphasise the need for the adoption of adaptive traffic assignment techniques to appropriately model the acquisition of information on a road network.

## Introduction

Traffic congestion is a problem affecting most metropolitan areas throughout the world resulting in significant economic, social and environmental costs. Alleviating congestion has been, and is still, a major focus for traffic engineers and transport planners. An initial solution to the problem was investing in the construction of transport infrastructure, to provide increased capacity within the network, providing users additional routes to travel to their destinations. Braess in 1968 analytically presented that the construction of an additional link, which connects two alternative routes between an origin and destination pair, may increase the travel cost for all users of the network [1]. The emergence of the Braess Paradox and other capacity paradoxes (such as the Down-Thomson Paradox [2]) has forced transport professionals to consider additional measures, other than increasing road capacity, to reduce traffic congestion. One approach is to focus on traffic management practices to improve resource utilisation, in an attempt to avoid congestion events. The provision of information to network users has been a recent initiative through the advent of GPS technology and the development of Intelligent Transportation Systems. In particular, Advanced Traveller Information Systems (ATIS) have been implemented to provide travellers with information about network conditions. The intention of these systems is to reduce the uncertainties of travel and as a result improve decision making by the user, benefitting the user as well as the system as a whole. For example, travellers can be informed of disruptions on the road network; those affected by the disruption may decide to alter their route choice, saving time at an individual level, potentially leading to a positive system wide impact.

Chorus, Molin [3] presents a detailed review of a number of analytical and simulation based research efforts that have investigated the impact of information on congested networks which support the notion that information provision improves performance. However, there have been a few studies suggesting that the provision of incomplete or imperfect information regarding route capacities and travel times could lead to a deterioration of performance [4–7]. Ben-Elia and Avineri [8] echoes these sentiments through a review of behavioural research associated with travel information. Though information can assist individuals in coping with uncertainty, the benefits on a network wide scale are debatable. Researchers have proposed potential adverse effects that can be a result of information provision as follows [5, 8–11];

- *Oversaturation*: The volume of information exceeds the cognitive capabilities of the individual resulting in decision making that does not correlate with the information provided.
- *Concentration and Overreaction*: Travelers all acquire information and make identical decisions, thus moving congestion from one location of the network to the other.

Extending these previous studies, this paper empirically investigates an analytical paradox where the provision of online information deteriorates road network performance through a controlled laboratory experiment. Recently, laboratory experiments have been used in studying driving behaviour as well as some specific equilibrium models and paradoxes [12–16]. In addition, experiments have defined the impact of information on road user risk attitudes [17], provided an understanding of the impacts of transport policy [18, 19] as well as studying the interaction of drivers at signalized intersections [20], highlighting its methodological value. A complete review of the application of experimental economics within the field of transport planning and engineering is detailed in Dixit, Ortmann [21] highlighting the strengths and weaknesses of the approach.

During the past decade a number of controlled laboratory experiments have been conducted on the traditional Braess Paradox [22–25] and the Downs Thomson Paradox [26]. Rapoport et al. presented the results of experiments investigating the Braess Paradox [23–25]. These studies involved simulated route choice games using networks susceptible to the Braess Paradox in a controlled laboratory environment. Rapoport, Mak [25] and Rapoport, Kugler [24] confirmed the presence of the Braess paradox and suggested that with learning and experience the paradox would be further exacerbated.

There have been two experiments that have specifically investigated the impact of pre-trip information on route choice [6, 27]. Both these studies investigated decisions in a controlled laboratory environment utilizing networks with two alternative congestible routes which vary unpredictably. Knorr, Chmura [27] discusses the role of pre-trip information as well as learning when selecting between a tolled route and an un-tolled route. The main finding of the study indicated that the provision of information to the entirety of the demand does not yield significant benefits to any individual traveller. This study provides some insightful contributions regarding the role of pre-trip information. However, in contrast to the research presented in this paper, the work by Knorr, Chmura [27] did not focus on any paradoxical scenarios. Rapoport, Gisches [6] presents an experiment designed to examine the work of Lindsey, Daniel [28]. The analytical model proposed by Lindsey, Daniel [28] is based on the classical “two-route network” model [29, 30]. The paradox suggested; “if free-flow costs on the two routes are unequal, travel cost functions are convex, and capacities are positively and perfectly correlated, then in equilibrium, paradoxically, total expected travel costs increase with the provision of pre-trip information about travel conditions on each route” [6]. Participants of the Rapoport, Gisches [6] experiment undertook a route choice task under two separate treatments, one without the provision of information and another with the provision of pre-trip information regarding the travel conditions of each of the routes. Each route had two cost functions, depicting “good” and “bad” conditions and this was the information provided to participants prior to each round of the “with information” treatment. The findings of the study were consistent with the paradoxical findings of the theoretical model presented by Lindsey, Daniel [28] suggesting that information is detrimental when conditions on both routes are perfectly correlated whilst information is beneficial when uncorrelated.

Probably the most relevant research to this study is the investigation into real-time online information by Lu, Gao [31] and the follow up study by the same team presented in Lu, Gao [32]. Similar to this study, Lu, Gao [31] conducted an experiment where participants faced a 3 route network with the possibility of an incident occurring on one of the routes. As with this study, treatments with and without descriptive online information provided at a downstream node were assessed to understand the impact of information on route choice. In addition, the impact of providing information about past performance of the entire network, “the foregone payoff” was also investigated. The findings show that information reduces the overall network travel time and increases travel time reliability. However, benefits of information provision depreciate when details of the foregone alternative is revealed to participants. The follow up study conducted in 2014 considered a greater sample size and also considered the case of providing feedback on subsequent rounds regarding only the chosen alternative. Similar positive results were obtained with the provision of information. It is interesting to note that 90% of the participants chose to travel on the paths that would provide them information of network conditions. This suggests that the concentration and overreaction phenomena may manifest, as it is clear that travellers desire information within a road network, creating impetus for the investigation presented in this paper. In addition to studying a specific analytical paradox where information deteriorates travel conditions, there are notable differences in the experimental methodology between this study and the studies conducted by Lu, Gao [32]. Specifically, this experiment involved incentivisation schemes based on the concepts of experimental economics.

It is clear that the implementation of information systems affects the behaviour and travel patterns of road users. Lately, there have been a number of simulation based studies centred on traffic flow theory which have revealed the potential positive and negative impacts of adaptive routing behaviour in light of disruptions and information on road network performance [33–36]. Furthermore, it is essential for planning models to account for the behavioural implications of information provision, which has led to a number of research efforts [37–41]. A static equilibrium model which incorporates the impact of online information on traffic assignment is User Equilibrium with Recourse (UER) [41]. UER advances the concept of traditional User Equilibrium (UE) to consider en-route decision making in light of information sources within a network. Unnikrishnan and Waller [41] present the analytical existence of a paradox, henceforth termed the “Online Information Paradox” (hereafter, OIP), where the provision of information for certain road networks increases the total system travel cost, questioning the viability of the information system. This paradox has only been presented theoretically [41] and it is essential to investigate the phenomenon in a physical setting. Similar to past studies, the occurrence of the paradox in a controlled laboratory experiment should implore transport planners and engineers to further evaluate the costs and benefits of en-route information for managing transportation networks, highlighting the importance of the research.

Accordingly, the focus of this study is to investigate the OIP presented in the UER framework. An experiment involving an incentivized computerized route choice game was designed to understand the route choice behaviour of participants. Two information regimes were considered: a base level treatment where participants are not provided any online information, defined as the “No Information” case and a scenario where participants received full perfect online information, defined as the “Information” case. Empirical data related to path utilization and total system travel costs were extracted from the experiment to compare with theoretical equilibrium solutions and also identify the presence of the paradox in a physical setting.

## The Online Information Paradox (OIP): Experimental design

### Origins of OIP: User Equilibrium with Recourse

Traditional equilibrium traffic assignment models, such as deterministic user equilibrium (UE), assume that users deterministically choose minimum cost paths, and then remain on that path regardless of realised network conditions. Accordingly, these approaches do not account for adaptive behaviour. In contrast, User Equilibrium with Recourse (UER) incorporates road users’ en-route decision making in the presence of information regarding the state of the network. The reader is referred to the paper by Unnikrishnan and Waller [41], where UER is introduced and discussed in detail, including mathematical formulations; this work contains only a brief description to assist in presenting what is described as a “Online Information Paradox” (OIP) observed within the equilibrium model.

UER is a static equilibrium model that accounts for one-step local information and user recourse on account of gaining that information [41]. An important assumption in the formulation is that the link cost functional forms are known but the link capacities are uncertain prior to departure. Only upon reaching an upstream node does a user begin to gain information about the capacity state of the following link. Thus, in a UER scenario, each link of a network could possess multiple “traffic states” with a probability of occurrence. Depending on the state of the link, which is disseminated through a source of information (Variable Message Sign, ITS technology), a user would choose the next link to travel on to reach his or her destination. To account for users’ response to different traffic states, UER considers a selection of possible hyper-paths known as routing policies instead of the least cost path [42].

Consider a network where a single main thoroughfare bifurcates into two possible paths to reach the destination, an arterial road and a freeway, and the paths have two possible states, state 1 and state 2. There are four possible routing policies in this case, users may always select the freeway regardless of the state of the network, or they may always select the arterial, or they may select the freeway if the network is in state 1 and the arterial if it is in state 2 or vice versa. Online information provided at the point of bifurcation regarding the state of the network only affects the class of users that will change their path en-route depending on the state of the network. Accordingly, a network considering user recourse is in equilibrium when, *“the expected cost of all used routing policies is minimum and equal and no user can unilaterally change their routing policies to improve the experienced expected cost”*[41, 42].

Unnikrishnan and Waller [41] provide a convex mathematical program for UER considering two scenarios. The first representation (defined as Model A) assumes that users that arrive at an upstream node observe the same link states for all outgoing links, meaning that system realisations (combinations of link realisations) are static over the period of analysis. The second representation (defined as Model B) assumes that users see different link states; therefore system realisations vary during the period of analysis. The experiment formulated considers the assumptions of Model A which reflects the scenario of a major disruption affecting the network for an extended period of time. In Model A, if the cost functions have a symmetric Jacobian and are strictly increasing, the formulation will have a unique solution. Unnikrishnan and Waller [41] presented that UER generally results in lower travel times than traditional equilibrium approaches emphasising the benefit of information provision, however, the paper also presents a paradox (OIP), considering a set of specific link costs and states, where information provision deteriorates network performance. A similar example is used as the context for the experimental design.

### Experimental context

**Fig 1:**
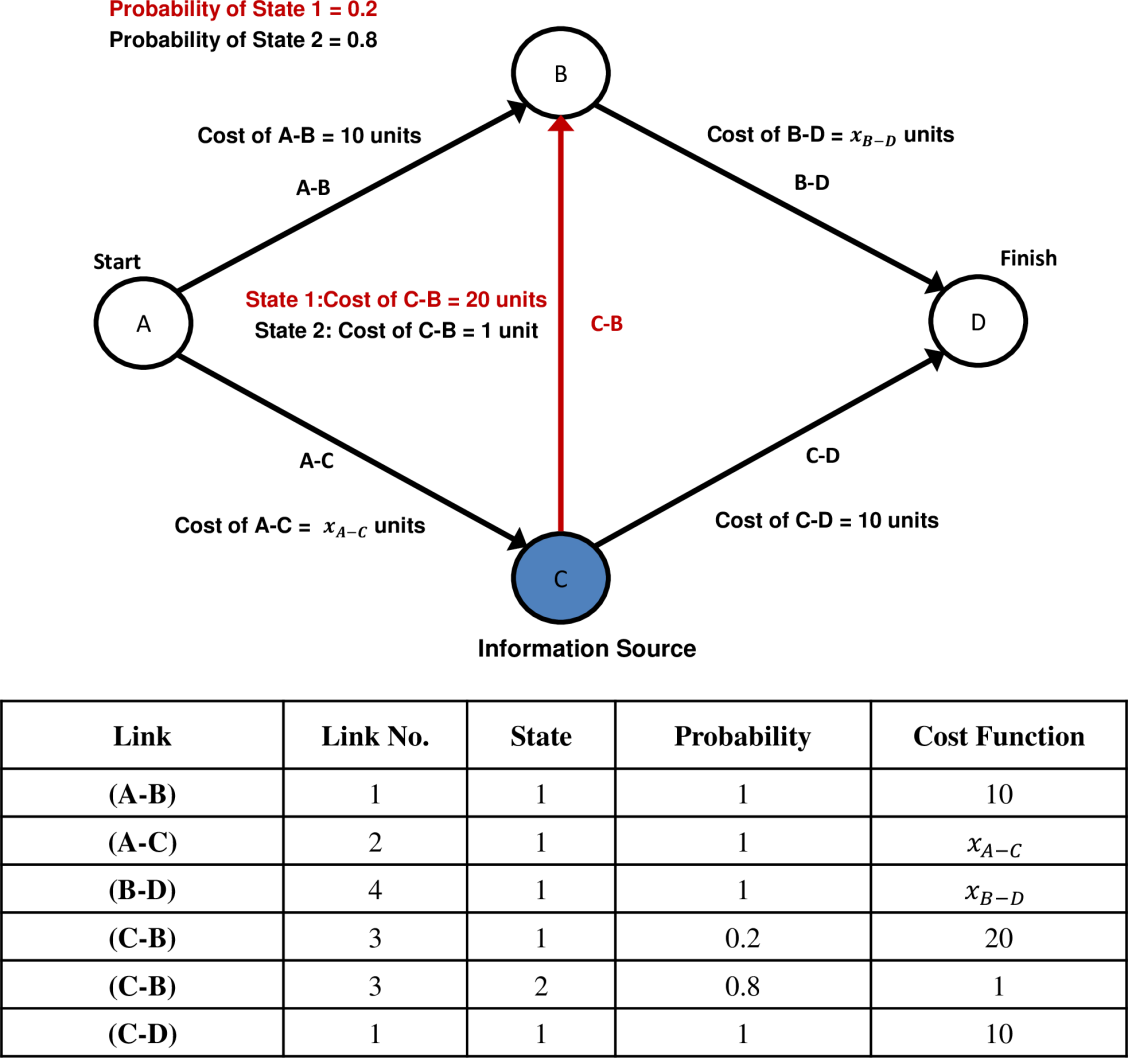
Online Information Paradox Network: Link states and respective cost functions.

The treatments of the experimental design were based on the following example of the OIP. Fig 1 presents the network, link states and the associated link costs.

The network services 12 units of demand travelling between origin node *A* and destination node *D*. Users travel along one-way links with the possibility of using three competing routes, *A-B-D*, *A-C-B-D* and *A-C-D*. If a user travels on link *A-B*, there is no option to alter the route in reaching the destination *D*. However, if a user selects link *A-C* there is the possibility of using link *C-B* and *B-D* to reach *D* or using link *C-D* to directly reach *D*. Thus, the users who initially selected link *A-C* can adapt their route depending on the prevailing traffic conditions of link *C-B* or link *C-D* if they are provided information at node C. The paradox compares the results of the User Equilibrium with Recourse traffic assignment model with and without the presence of perfect information. The information provided at node *C* is related to the state of link *C-B*, where 20% of the time the cost of using link *C-B* is equal to 20 units (State 1) and the remaining 80% of the time the cost of using link *C-B* is equal to 1 unit (State 2). All other links within the network have a single state with no variation in cost functions as presented in Fig 1. This scenario is analogous to road *C-B* experiencing a disruption such as an accident or breakdown during State 1 which results in the closure of lanes and the increase of travel cost for all users.

### Theoretical equilibrium conditions

The theoretical equilibrium conditions were calculated to compare the results of the experiment using the following models;

- Expected User Equilibrium (EUE);
- User Equilibrium with Recourse (UER);

The EUE approach is similar to the traditional User Equilibrium (UE) approach as it considers the minimization of all utilized path travel costs [43]. However, instead of considering separate equilibrium conditions for each traffic state, EUE considers the expectation of link costs to account for the uncertainty. This model assumes that users experience the different states of the network; however instead of having perfect knowledge of the realization of each state, the users develop an understanding of the average travel conditions. It is important to note that in general transport users are assumed to be risk neutral. In relation to the assessment of the OIP, EUE serves as a representation of the no online information scenario, where users cannot adapt en-route. Similar to traditional UE, EUE accounts for day to day experiences resulting in long term equilibrium conditions. Travellers on link *A-C* are not aware of the traffic conditions of either link *C-D* or link *C-B* and accordingly consider expected costs to determine the minimum cost path. Thus, under the no online information case, for 12 units of demand, the EUE solution decomposes to the UE solution where demand is split evenly across path *A-B-D* and *A-C-D*, assuming expected link costs. The EUE solution is presented in Table 1.

**Table 1:** Analytical equilibrium conditions: Expected User Equilibrium (EUE) traffic.

EUE Conditions (No Information)		
Path	Flow	Path Cost
**A-B-D**	6	16
**A-C-B-D**	0	16.8
**A-C-D**	6	16
**Total System Travel Cost (TSTC)**		**192**

NB: Under EUE conditions, 6 units of demand travel on path A-B-D and 6 units of demand travel on path A-C-D with all users experiencing an expected path cost of 16 units which consequently results in a TSTC of 192 units.

In order to determine the equilibrium solution for the “Information” case, the UER solution is calculated [41]. The five routing policies which an individual would consider in this example are:

- Policy 1 (P1): State 1 Path: **A-B-D**, State 2 Path: **A-B-D**
- Policy 2 (P2): State 1 Path: **A-C-B-D**, State 2 Path: **A-C-B-D**
- Policy 3 (P3): State 1 Path: **A-C-B-D**, State 2 Path: **A-C-D**
- Policy 4 (P4): State 1 Path: **A-C-D**, State 2 Path: **A-C-D**
- Policy 5 (P5): State 1 Path: **A-C-D**, State 2 Path: **A-C-B-D**

The core assumption of this traffic assignment technique is that all users arriving at an upstream node observe the same link states for all outbound links. This means that the state of each link is static throughout the assessment, for the entirety of the demand traversing the network. This is parallel to an incident disrupting traffic for a period of time or the presence of sustained peak period congestion in a portion of a network. The UER solutions calculated for this example indicate that 4 units of demand select *P1*, 3 units select *P4* and 5 units select *P5*, as shown in Table 2.

**Table 2:** Analytical equilibrium conditions: User Equilibrium with Recourse (UER) traffic conditions.

UER Conditions (Information)									
Policy Statistics					Path Statistics				
Policies	State 1 Path	State 2 Path	Policy Flow	Policy Cost	Path	State 1 Flow	State 1 Cost	State 2 Flow	State 2 Cost
P1	A-B-D	A-B-D	4	18	A-B-D	4	14	4	19
P2	A-C-B-D	A-C-B-D	0	20.8	A-C-B-D	0	32	5	18
P3	A-C-B-D	A-C-D	0	20.8	A-C-D	8	18	3	18
P4	A-C-D	A-C-D	3	18					
P5	A-C-D	A-C-B-D	5	18					
**Total System Travel Cost (TSTC)**			**216**						

NB: Under UER conditions, 4 units of demand travel using policy P1, 3 units of demand travel using policy P4 and 5 units of demand travel using policy P5 with all users experiencing an expected path cost of 18 units, resulting in a TSTC of 216 units. The expected path cost is calculated by considering the state dependent flows of each path as shown in the path statistics of the table.

Comparing the no online information case (Table 1) with the information case (Table 2), it is clear that individual travel costs and system performance depreciates as a result of information provision. Travel cost for the individual increased from 16 to 18 and total system travel cost increased from 192 to 216 highlighting the caution required when implementing information systems within road networks.

### Experiment conditions

#### Computerized route choice game treatments

A computerised route choice game served as the core element of the experiment. The experiment was programmed and conducted using software specifically designed for the application of economic experiments, zTree [44]. The game replicated the network properties and demand conditions described in subsection 2.3. Participants were divided into groups of 12, where each participant represented a unit of demand. The participants played the role of motorists who repeatedly had to travel between a specified origin (*A*) and destination (*D*). As presented in the example, the participants had a choice of 3 routes, *A-B-D*, *A-C-B-D* and *A-C-D*. At the completion of each decision period, the traffic conditions experienced by all the participants were revealed to every participant prior to making the next routing decision. The state of the link *C-B* was determined randomly through zTree but satisfied the likelihood of occurrence of 20% for State 1 and 80% for State 2, this means that each group faced State 1 conditions in 4 out of the 20 periods for each treatment, as the network game was repeated across 20 rounds for each treatment (within subject design). The purpose of divulging individual and network performance of each journey was to capture any learning effects across both information regimes. Even though there is a unique equilibrium solution for both the no information (EUE) and information (UER) treatments, there are numerous ways the participants could arrange themselves to achieve the equilibrium solution. Take for example the no information case; the EUE solution indicates that route *A-B-D* and route *A-C-D* will contain 6 units of demand each. The participants have 924 (C(12,6)) different combinations of achieving this flow pattern presenting a very complex coordination problem as participants only have access to historical travel conditions [6, 15, 24]. Based on the difficulty of the task, achieving equilibrium flow patterns on average is a significant observation. In order to investigate the presence of the OIP, two treatments were carried out:

- ***Treatment 1***: No online information provided
- ***Treatment 2***: Online information provided at Node C regarding the prevailing traffic conditions on link C-B

Participants traversed a network that was similar to Fig 1. The only difference being that instead of referring to the traffic state of link *C-B* as “State 1” and “State 2”, the route selection was placed in the context of an incident occurring on link *C-B*. State 1 referred to “Incident” conditions and State 2 referred to “No Incident” conditions on link *C-B*. This contextualisation was undertaken to place participants in a realistic driving scenario that they could face on their daily commutes. The method in which participants selected a route varied based on the treatment. Treatment 1, involved no provision of online information, this meant that each participant selected the entirety of the route from origin *A* to *D*. Treatment 2 included the provision of information at node *C*, as a result participants performed the route selection in a staged manner on a link by link basis.

#### Additional tasks and incentive structure

Additional tasks were undertaken to gather information of the characteristics of each of the participants to better understand individual decision making. After completing the route choice tasks, participants were asked to fill out a short 10 minute socio-demographic questionnaire. It must be emphasised that the focus of this particular paper was the observation of the OIP at an aggregate level and as such the analysis of additional task data will not be detailed within this paper.

A key aspect of experimental economics is incentivisation to ensure that the participants’ decisions had realistic consequences. Incentives for this experiment involved a static participation fee of AUD$5 and performance based reward surrounding the multiple price lottery and each of the computerised route choice tasks. In terms of the route choice tasks, each participant received an income of 45 units for each of the periods of both tasks. The payoff for a round was calculated by deducting the travel cost experienced by the participant from the initial income. At the end of both treatments, a random period from each task was selected and the payoff associated with that round was paid to the participant, where every unit equaled AUD$0.25. On average, a participant could earn approximately $25 across the session.

The route choice tasks involved repeated decision making which can potentially result in participant fatigue, affecting decision making and ultimately the results of the experiment. Accordingly, a pilot test was conducted to assess the number of feasible periods that could be played by a participant. A group of 12 colleagues from the Research Centre for Integrated Transport Innovation at the University of New South Wales (UNSW) participated in the pilot study and were asked to provide feedback on the length of the experiment and comment on how many periods should be put in place. The pilot study was conducted over 2 hours, where 20 rounds of each task were completed. The participants revealed that this length of time and number of periods neared the limits of their cognitive capabilities and that anymore periods would be a strain for the participant. It is acknowledged that the number of periods used for this experiment is significantly lower than that conducted recently by Knorr, Chmura [27] and Rapoport, Gisches [6], which used 50 rounds per treatment and 80 rounds per treatment respectively. However, these previous experiments involved a decision between 2 routes as compared to the 3 routes considered in this study, reducing the cognitive load on the participant. Furthermore, the information task involved staged decision making based on the acquisition of online information which increases the time taken to complete each round. Based on these key differences, and the findings of the pilot study it was decided that the participants should undertake 20 periods of each treatment.

#### Recruitment process and experimental procedure

Participants were recruited using the UNSW Australian School of Business (ASB) Experimental Research Laboratory. Admission to participate in the study was contingent on respondents being at least 17 years old to ensure that participants understood the meaning of route choice in a real driving scenario. The experiment was approved by the UNSW Australia Human Research Ethics Advisory panel and each participant signed informed consent forms signifying their willingness to participate. The laboratory used for the experiment contained computer workstations with enough space between participants to prevent collusive activities. Each session consisted of a total of 24 participants who were randomly separated into 2 groups of 12 participants to complete the route choice tasks. The procedure of the experiment can be summarized as follows;

1. Participants completed the computerised route choice tasks across both Treatment 1 and Treatment 2.
2. Participants completed the demographic questionnaire.
3. Participants’ payoffs were calculated and each participant was paid.

To control for order effects the sequence of the treatments were varied across the sessions. The experiments with the following sequences of tasks were conducted across different sessions (a complete set of Experiment Instructions for Order 0 are provided in: ‘S1 Appendix. OIP Experiment Instructions’).

- Order 0: Treatment 1 (No Information, 20 periods), Treatment 2 (Information, 20 periods), Questionnaire.
- Order 1: Treatment 2 (Information, 20 periods), Treatment 1 (No Information, 20 periods), Questionnaire

Six sessions of the experiment were held during September and October 2015 (Sessions 1 to 3 undertook Order 0, while Sessions 4 to 6 undertook Order 1 described above). Each session involved 24 participants thus obtaining data from a total of 144 participants. Table 3 presents a summary of the key demographic characteristics of the participants, there was a balance between genders with 47% of the participants being male and 53% being female. More than 68% of the participants had greater than one year’s experience in driving a vehicle suggesting that there was a certain level of understanding in relation to route choice and driving. As the participants were recruited from a University staff and student pool, it is acknowledged that the average age of the participants is young at 22.14 years (age range: 18 to 40). However, it should be noted that 22% of the participant pool were above the age of 24 indicating that this study did not focus exclusively on undergraduate student behavior, obtaining a degree of diversity.

**Table 3:** Summary of demographic characteristics of participants.

Characteristic	Values
Number of Male Participants	67
Number of Female Participants	77
Driving Experience (>1 year, < 1 year)	98/46
Average Age	22.14
Range of Ages	18–40

## Results and analysis

Each round of the computerized route choice game provided a traffic assignment pattern (1 observation) which could be compared with the theoretical equilibrium results. Since each session consisted of two groups of 12 participants a total of 40 observations were recorded for each treatment. Therefore, across the six sessions conducted, there were 240 observations for each treatment. A complete set of the data collected from the experiment is provided in: ‘S2 Appendix. OIP Experiment Data’. The data obtained was statistically analyzed to compare with the predictions of the theoretical models and identify the presence of the OIP. The key performance metrics assessed were path utilization across the three routes and the Total System Travel Cost (TSTC) which describes the system wide performance of the network.

Table 4 presents a comparison of the analytical path flow solutions calculated in subsection 2.3 and the observed mean path flow results from the experiment. The EUE solution depicting the “No Information” case suggested that there will be a 50% split of the demand on route *A-B-D* and *A-C-D*, and zero usage of *A-C-B-D*. The observed mean flows differ from this solution, with a mean flow of 1.788 on path *A-C-B-D*. On average, across the entire data set there were 1 to 2 participants who selected *A-C-B-D*. This can be explained by individuals hoping that State 2 conditions would prevail and they would be able to significantly reduce their travel cost by encountering the 1 unit cost on link *C-B*. This tends to suggest that the EUE solution is not capturing the behavior of users in the no information case. However, it should be noted that across the last 3 periods of Treatment 1, participants learnt from the experiences of the previous rounds and there was indication that the data may converge to the EUE solution if more periods were considered. The UER solution provides the theoretical representation of Treatment 2, where participants are provided online information. UER solutions are presented in terms of routing policy usage and as such the path flows need to be disaggregated for each state of the network. Comparing the observed state based mean path flow from the experiment to the theoretical solution indicates that the UER solution closely depicts the empirical behavior. The rounded integer values of each of the observed mean path flow values match exactly with the theoretical UER solution.

**Table 4:** Comparison of empirical and theoretical path utilization.

Treatment 1: No Information					Treatment 2: Information provided at Node C				
	State	A-B-D	A-C-B-D	A-C-D		State	A-B-D	A-C-B-D	A-C-D
EUE Solution	E(S1,S2)	6	0	6	UER Solution	S1	4	0	8
Observed Mean Path Flow	S1	4.896	2.188	4.917		S2	4	5	3
	S2	5.130	1.688	5.182	Observed Mean Path Flow	S1	4.292	0.146	7.563
	Overall	5.083	1.788	5.129		S2	4.286	4.828	2.885

Table 5 displays a comparison of TSTC between treatments. The results of the experiment supports the presence of the OIP, as the mean TSTC increases from 210.629 to 219.163 cost units when information is provided to the participants. However, the difference in mean TSTC between the two treatments is less than what was predicted in theory. The analytical solution suggests a difference of 24 cost units far greater than the 8.534 units observed on average during the experiment. This can be attributed to participants using *A-C-B-D* in the no information scenario resulting in a higher average travel cost for the system and straying away from the EUE solution. However, by observing the latter periods of the data set, it is clear that the difference begins to reflect the theoretical predictions. The mean TSTC of the last 3 periods for the no information treatment is 194.833 units, while for the information treatment it is 219.333 units, again highlighting the initial periods of exploration carried out by the participants.

**Table 5:** Comparison of mean travel costs between treatments.

	Treatment 1: No Information	Treatment 2: Information Provided at Node C	
State	E(S1,S2)	S1	S2
**Cost of A-B-D**	16.871	14.438	19.115
**Cost of A-C-B-D**	18.588	32.146	17.828
**Cost of A-C-D**	16.917	17.708	17.714
**Observed TSTC**	**210.629**	**219.163**	
***σ_TSTC_***	24.948	15.470	
**Theoretical TSTC**	192	216	

In order to corroborate the observed empirical presence of the OIP, a paired T-test was completed to determine if the mean TSTC values were significantly different between the treatments. If the T-statistic exceeds the T-critical value there is a significant difference between the observed TSTC values. Table 6 clearly shows that when assessing the period data, the presence of the OIP is statistically significant. The period data used for the statistical analysis contained repeated measures of the mean TSTC for each group of participants across both treatments. For example, group 1 contains 20 measures of mean TSTC, under each information regime. Thus, to account for repetition, a Wilcoxon Signed-rank test was used to compare the group mean TSTC statistics. The Wilcoxon Signed-rank test was used due to the small sample size relaxing the assumption of normality present in the paired T-test. To further emphasize the evidence, 11 out of the 12 groups (greater TSTC highlighted in orange within Table 6) within the experiment presented a statistically significant OIP at a confidence level of 95%. A Mann Whitney U-Test was also conducted using the group data to identify order effects. The group data was separated by treatment and the 6 observations of each order were compared, p-values exceeded 0.13, indicating no order effects and highlighting the consistency of the experimental procedure. These findings suggest that presence of the OIP is highly significant.

**Table 6:** Statistical analysis considering periodic data and group data.

**Period Analysis: Paired T-test (240 observations of TSTC)**						
		**Treatment 1: No Information**			**Treatment 2: Information Provided at Node C**	
***μ_TSTC_***		210.629			219.163	
***σ_TSTC_***		24.948			15.470	
**T-statistic**		4.53811				
**T-critical**		1.96994				
**p-value**		0.00001				
**Group Analysis: Wilcoxon Signed-rank Test (12 observations of mean TSTC)**						
		**Treatment 1: No Information**		**Treatment 2: Information Provided at Node C**		
**Session Number**	**Group Number**	**Mean TSTC**	***σ_TSTC_***	**Mean TSTC**		***σ_TSTC_***
**1**	**1**	212.200	26.411	220.300		13.739
**1**	**2**	209.650	25.887	218.600		12.701
**2**	**3**	209.800	19.259	215.800		11.181
**2**	**4**	210.550	21.333	220.600		14.233
**3**	**5**	217.500	36.699	215.900		10.228
**3**	**6**	212.600	34.014	229.150		26.650
**4**	**7**	213.850	35.445	216.400		14.591
**4**	**8**	208.750	16.676	215.800		15.477
**5**	**9**	204.700	15.815	220.800		17.025
**5**	**10**	211.850	20.254	217.600		13.068
**6**	**11**	205.350	16.925	219.100		13.082
**6**	**12**	210.750	22.583	219.900		16.613
**W-statistic**		1				
**W-critical**		17				
**p-value**		0.000317				

Participant learning questions the statistical analysis suggesting the empirical existence of the OIP, as data from all 20 periods of the experiment were analyzed collectively. It could be argued that the emergence of the paradox is an artefact of the data set. However, given that both Treatment 1 and Treatment 2 indicated that participants were learning and converging towards the analytical solutions, it is clear that upon learning, the manifestation of the OIP will most likely be stronger than what is presented within the aggregate analysis in this paper.

Another interesting observation from Table 6 is the difference in the standard deviation of the TSTC between the information scenarios. Intuitively, there is a far greater standard deviation in costs when no online information is provided, as participants face greater rewards and losses associated with selecting route *A-C-B-D* and the uncertainty of the cost of link *C-B*. This contrasts the situation presented in Treatment 2, as individuals are able to adapt if State 1 of link *C-B* eventuates and there is an incident. The result suggests that though information has the potential to increase TSTC, there is a reduction in the variance of the travel costs observed within the system and as a result offers a greater degree of reliability.

**Fig 2:**
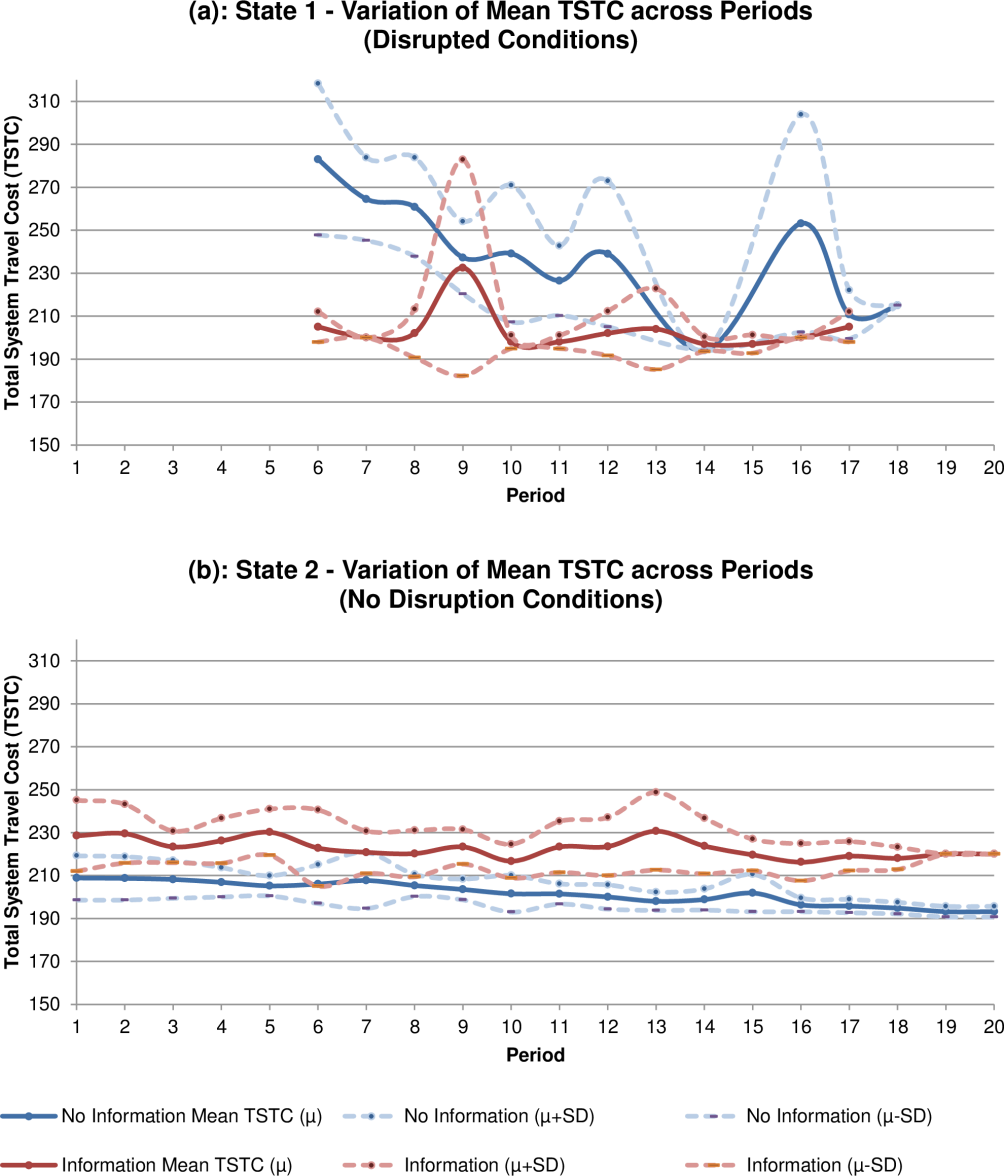
**Variation in Mean TSTC Across Periods (a) State 1: Disrupted Conditions, (b) State 2: No Disruption Conditions (normal conditions)**.

Fig 2 further emphasises how information can be beneficial in disrupted conditions and its potential to maintain a greater degree of reliability. The graphs in Fig 2 present the variation in the mean and standard deviation of TSTC across the periods of the experiment separating “State 1” and “State 2” conditions (Fig 2(A) and 2(B) respectively). Disrupted conditions in Fig 2(A) shows volatility in TSTC and also highlights the value of information. Under these State 1 conditions, the mean TSTC is generally less and considerably more stable for the information scenario as compared with the no information scenario. In the “No Disruption” case, or normal traffic conditions (Fig 2(B)), there is far greater stability in TSTC values across both information and no information scenarios and the provision of information consistently deteriorates network performance. Therefore, the likelihood and frequency of disrupted conditions must be accounted for prior to the dissemination of online information as it is quite evident that benefits arise in scenarios of greater uncertainty.

## Discussion and limitations

The results of the experiment indicate the empirical presence of the Online Information Paradox, where the provision of online information deteriorates network performance and increases the travel costs for users. Furthermore, travel time variability reduces with the presence of information, consistent with the observations made by Lu et al. [31, 32]. This is evident as the standard deviation of TSTC in the information case across all assessments was considerably lower than that of the no information case.

It is also important to emphasise that online information provision, in general, will reduce travel costs and improve the efficiency of a network at an analytical and empirical level as presented in a number of studies [31, 32, 45, 46]. However, what is evident from this study and that by Rapoport et al. in 2014 is that for specific network topologies, there are situations where the provision of information can deteriorate network performance. Accordingly, based on the theoretical presentation of the UER framework and the corroborating empirical evidence, careful consideration of network structure is essential to avoid the emergence of such a paradox when evaluating the implementation of ATIS and ITS systems within road infrastructure.

The authors acknowledge that there are limitations within this study. The fundamental abstraction from reality is that travel cost was the only criteria used by participants in the route selection process. Route choice decisions are in general multi-objective where travelers consider travel time, reliability, tolls, road hierarchy and other factors [47]. The network and costs functions used to assess the paradox within the experiment are simplifications of reality. However, there is justification for this approach in an experimental as well as a cognitive context. In order to ensure control and reliability within an experiment, each participant must be able to comprehend the task to gain valuable insights [21]. In addition the simplification can be considered realistic as individuals cannot acquire as well as process all alternatives completely. Naturally, users reduce choice sets and simplify complex cost structures by evaluating the most important components [48, 49].

## Conclusion

The aim of this study was to investigate the empirical presence of a theoretical transportation paradox, defined as the “Online Information Paradox”. The paradox suggests that the provision of online information deteriorated the travel conditions for individual users as well as the system as a whole. The analytical presence of the paradox was derived from two equilibrium models; the Expected User Equilibrium (EUE) solution explained the case where individuals did not have access to information while the User Equilibrium with Recourse (UER) solution depicted the case of online information dissemination at an intermediate node between origin and destination. Concepts of experimental economics were used to develop a controlled incentivized laboratory experiment consisting of a repeated computerized route choice game. The game emulated a 3 route stochastic network with an intermediate node that allowed a user to swap routes. The stochasticity of the network was dependent on the presence of an incident on a single link of the network with a given probability of occurrence.

Aggregate statistics of path flow and Total System Travel Cost (TSTC) were used to compare the empirical findings with the theoretical findings. A total of 12 groups of participants completed the experiment and the Online Information Paradox was observed in 11 of the 12 cases and its presence across all the data was statistically significant at a confidence level of 95%. Though the OIP was observed, the presence of information resulted in significantly lower standard deviations of system travel costs supporting the claim that information improves travel time reliability.

The observation of the Online Information Paradox in a controlled setting, as presented in this study, creates an additional consideration for transport authorities during the evaluation and implementation of ITS and ATIS infrastructure within a road infrastructure context. Replications of such experiments and more importantly field based identification of the phenomena will require transport professionals to be aware of the paradox. Finally, the study indicates that practitioners should consider adaptive traffic assignment techniques, such as UER, to appropriately model the acquisition of information on a road network.

## Supporting information

S1 Appendix OIP experiment instructions. This document provides instructions for Order 0 of the Experiment. (N.B. Order 1 includes identical wording with a reordering of the treatments).
(PDF)

S2 Appendix OIP experiment data. This spreadsheet provides all data collected from the experiment.
(XLSX)
