# Supplementary material for: An experimental study of the Online Information Paradox: Does en-route information improve road network performance?
Source: PLoS One. 2017 Sep 13;12(9):e0184191. doi: 10.1371/journal.pone.0184191 (PMC5597203; doi:10.1371/journal.pone.0184191)
Supplement: S1 Appendix — This document provides instructions for Order 0 of the Experiment. (N.B. Order 1 includes identical wording with a reordering of the treatments). (PDF) [file pone.0184191.s001.pdf]

Welcome to the research study.

Please wait for an assistant to get you started.

PLEASE DO NOT WRITE ON THESE INSTRUCTIONS

Welcome and thank you for coming along to this experiment. During this experiment you'll be asked to make decisions a series of three choice tasks. The tasks give you the opportunity to earn money. You will be asked to make these decisions with the objective of maximising your payoff across the three tasks. Your payoff will vary from $0 to as high as $35 and will depend on the decisions you make as well as the decisions made by the other participants. In addition to this payoff, you will receive $5 for your participation throughout the session. You will be paid in cash today at the end of the session. In these tasks you should make decisions that seem right to you. All of your decisions will remain anonymous.

Please read these instructions on the following pages. In case you have any questions or concerns, please raise your hand and the experimenter will come to answer them.

Please note that hereafter communication between the participants is strictly prohibited. If there is any communication with one another in any shape or form, the experiment will be cancelled and no participants will receive any payment.

The session will contain an individual choice task, 2 route choice games and a short questionnaire.

Initially, you will complete an individual choice task where you must select between 2 competing options across 10 varying scenarios. The instructions for this task will be provided to you on the following page.

Within the games, you will be asked to serve as motorists and choose a route to travel from your origin (start) to your destination (finish). The instructions for Game 2 will be presented after completion of Game 1. You will play Game 1 and 2 for 20 identical rounds each.

At the end of the session you will be paid for each task that you complete. The payoff for the individual choice task will be a single round randomly selected for each of the 10 scenarios. Similarly a random round will be selected from the 20 rounds in Game 1 and Game 2 respectively. The payoff units from the 2 games will be multiplied by $0.25 and added to the payoff received in the individual choice task.

PLEASE DO NOT WRITE ON THESE INSTRUCTIONS

Individual Choice Task

The computer screen in front of you presents a table containing 10 choice scenarios. Each choice scenario involves you deciding between 2 options, option A and option B.

The first row indicates that option A gives you a 10% chance of getting $5 and 90% chance of getting $4, and that option B gives you a 10% chance of getting $9.75 and 90% chance of getting $0.25. Your task is to click on the option you prefer (A or B) for each choice scenario. When you have completed all 10 scenarios choosing between option A and option B, click on "confirm" to register these choices. Your choices can be changed as many times as you wish until you click on the confirmation button.

The payoff for this part will be determined in the following way. The central computer will pick randomly for each participant, one scenario, among the ten scenarios presented to you. Then, depending on the particular scenario selected by the computer, the computer will make an additional drawing lot in determining a number between 1 and 10. This number will finally determine your payoff for the individual choice task.

Let's take an example. Assume that the computer picked randomly for you, choice scenario 3, where you chose option A. In this situation, option A gives you a 30% chance to get $5 and a 70% chance to get $4. If the computer randomly chooses a number between 1 and 10 by the computer which is either 1, 2 or 3, you will get $5 and if the number is 4, 5, 6, 7, 8, 9 or 10, you will get $4. If for choice scenario 3, you chose option B, you will gain $9.75 if the number is between 1 and 3 and $0.25 if the number is between 4 and 10.

The payoff for the individual choice task will be part of your final payoff, as explained before.

Please raise your hand if you are unclear with the instructions.

Please wait for further instructions.

Do NOT turn over the page until instructed to do so.

PLEASE DO NOT WRITE ON THESE INSTRUCTIONS

Game 1

Consider the traffic network presented in the figure below. Each driver is required to choose a route in order to travel from the starting point, denoted by *A*, to the final destination, denoted by *D*. There are three alternative routes and they are represented in the diagram as [*A–B–D*], [*A–C–D*] and [*A-C-B-D*]. The number of drivers selecting each road, in other words the volume of traffic on each road, is denoted by "x_n" where n refers to the relevant road.

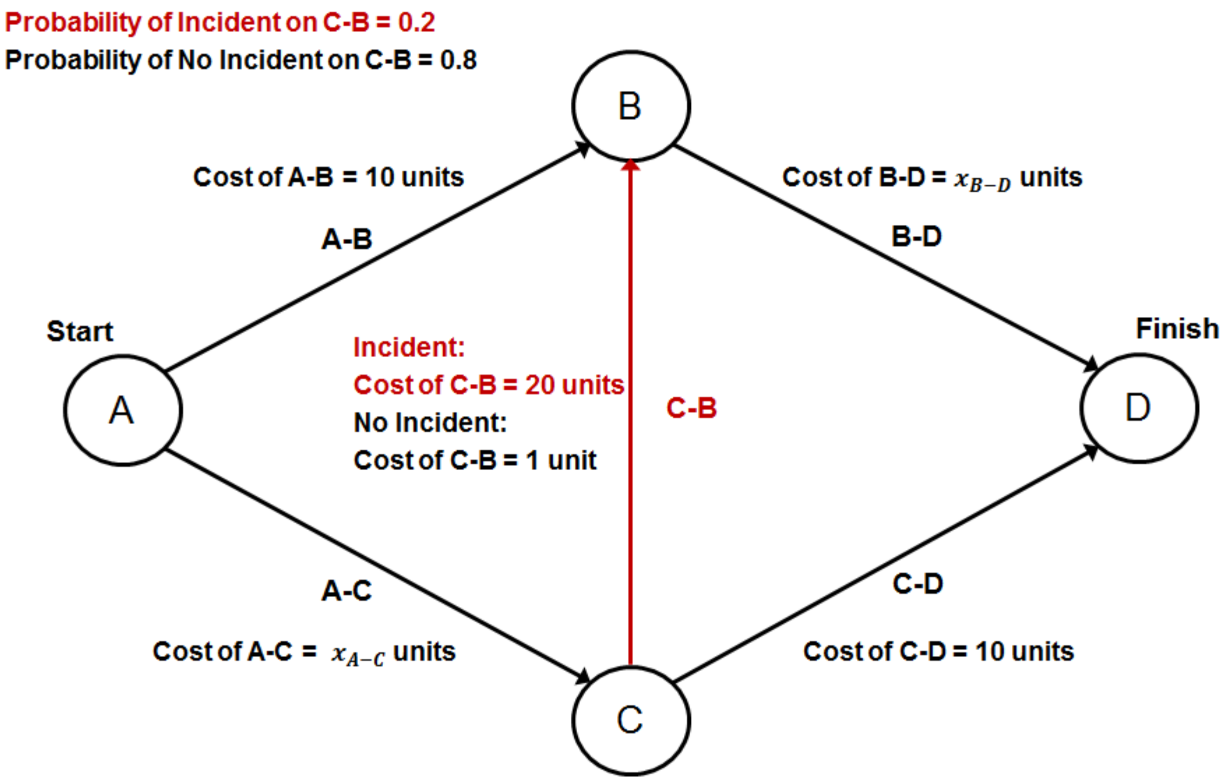

Figure 1 - Traffic Network of Game 1

Travel from an origin to a destination bares a cost for each individual, the cost of time, fuel and any tolls incurred along the route. In addition there are uncertainties when travelling, incidents may arise, weather may create difficulties in driving or there may be an excessive number of vehicles on the network due to peak travel conditions. These types of factors affect the state of the network and the travel costs for each road on the network.

In this particular network, the cost of traveling on each of the road segments (provided in "units") is written near the labels of the road segments. The costs of roads A-B and C-D are fixed at $10, while the cost of A-C and B-D are dependent on number of motorists travelling on the road segments, denoted by x_{A-C} and x_{B-D} respectively. The network describes a scenario where there is a **20% chance of an incident occurring on road segment C-B** which increases the fixed cost of road segment C-B from 1 unit to 20 units as the disruption increases travel time and fuel costs.

Your activity will be to select the route which you think would be the best to travel from node A to node D.

You will NOT know any information about the presence of an incident on C-B prior to making your route selection.

Procedure for Game 1

At the beginning of each round, the computer terminal in front of you will show you the network diagram, presented in Figure 1 on the previous page, containing the three possible routes described before; [*A*–*B*–*D*], [*A*–*C*–*D*] and [*A-C-B-D*]. The following steps are required to be carried out for each round.

1. You will be asked choose which of the routes you wish to travel on. To choose a route, click the roads related to the route. For an example if you choose route [A-B-D], click on road [A-B] and road [B-D]. Once you click on a specific road segment of a route, that segment will be highlighted in a red colour. If you make an error whilst clicking on a road segment you should either click an alternative road segment or click “Cancel” to restart the selection process. After you have connected origin A and destination D with a feasible route that you would choose to travel on, you will be asked to “Confirm” your route by pressing the “Confirm” pop-up button if you are satisfied with your selection.
2. After confirming your route choice, you will be presented with a message to wait until all other participants have made their decisions.
3. Upon the completion of the task by all participants the computer will present you with the following information;
    a. The traffic conditions of Link C-B: “No Incident on C-B” or “Incident on C-B”
    b. The route you have chosen and the payoff for the round
    c. The number of participants who chose route [*A-B-D*]
    d. The number of participants who chose route [*A-C-D*]
    e. The number of participants who chose route [*A-C-B-D*]
4. You will complete this activity for 20 rounds. At the completion of 20 rounds, the computer will inform you that Game 1 has been completed. A further message will be provided to “Please wait until the experiment continues”

All the decisions will only be made by clicking the “mouse” of the computer; there is no need to type anything whilst participating in the game.

You will receive **45 units** of income for each round. The payoff for each round will be determined by the following equation:

Payoff = (45 – Total Cost of travel) units

All 20 rounds will have the same structure.

At the end of both games (Game 1 and Game 2) you will receive payment for a randomly selected round from each of the games. You will receive **$0.25** for every unit of payoff earnt in the randomly selected rounds of each of the games.

Example of Travel Costs

Let's consider that in the first round you are the only one to choose route [A-C-B-D], 6 participants choose [A-B-D] and 5 participants choose [A-C-D]

- If road **C-B does NOT experience an incident**, the cost of using road A-C is 6 units (5 people using A-C-D and you are using A-C-B-D), the cost of using road C-B is 1 unit and the cost of using road B-D is 7 units (6 people using A-B-D and you are using A-C-B-D). Therefore your total cost will be (6+1+7) = **14 units** for that round. (Figure 2)
- If road **C-B experiences an incident**, the cost of using road A-C is 6 units, the cost of using road C-B is 20 units (fixed cost) and the cost of using road B-D is 7 units (6 people using A-B-D and you are using A-C-B-D). Therefore your total cost will be (6+20+7) = **33 units** for that round. (Figure 3)

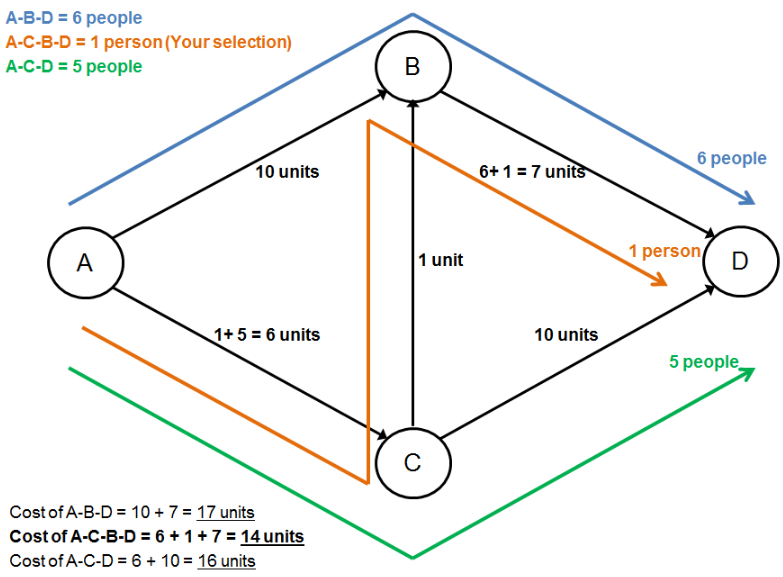

Figure 2 - Costs of Routes (No Incident on C-B), Round 1

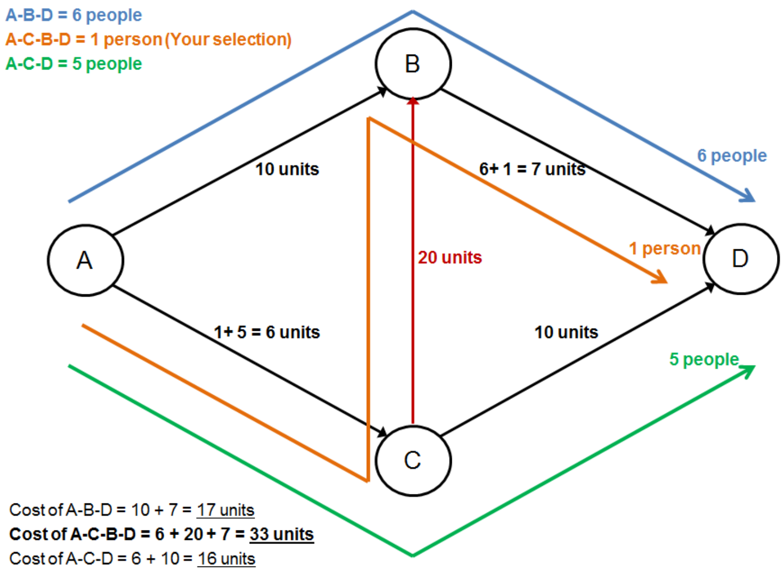

Figure 3 - Costs of Routes (Incident on C-B), Round 1

Now let's consider that in a subsequent round (Round 2) of the game you and 2 other participants choose route [A-C-B-D], 4 participants choose [A-B-D] and 5 participants choose [A-C-D].

- If road **C-B does NOT experience an incident**, the cost of using road A-C is 8 units (3 people using A-C-B-D and 5 people using A-C-D), the cost of using road C-B is 1 unit and the cost of using road B-D is 7 units (3 people using A-C-B-D and 4 people using A-B-D). Therefore your total cost will be (8+1+7) = **16 units** for that round. (Figure 4)
- If road **C-B experiences an incident**, the cost of using road A-C is 8 units, the cost of using road C-B is 20 (fixed cost) and the cost of using road B-D is 7 units. Therefore your total cost will be (8 + 20 + 7) = **35 units** for that round. (Figure 5)

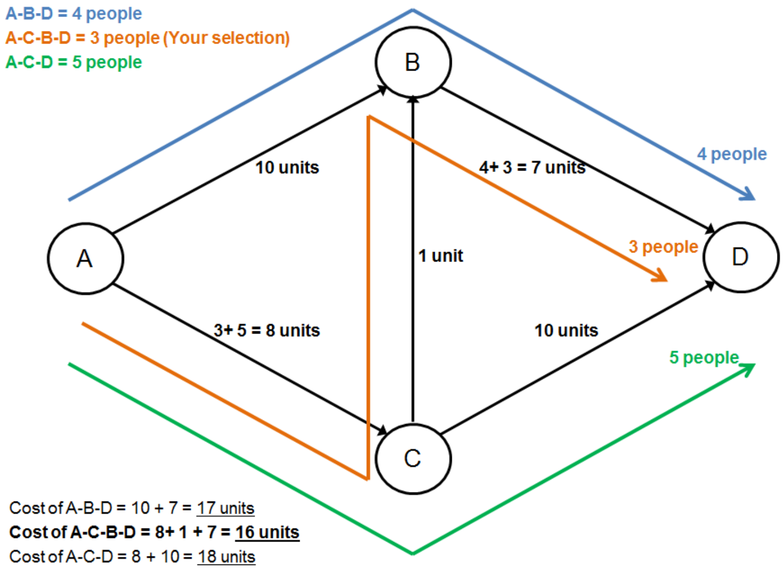

Figure 4 - Costs of Routes (No Incident on C-B), Round 2

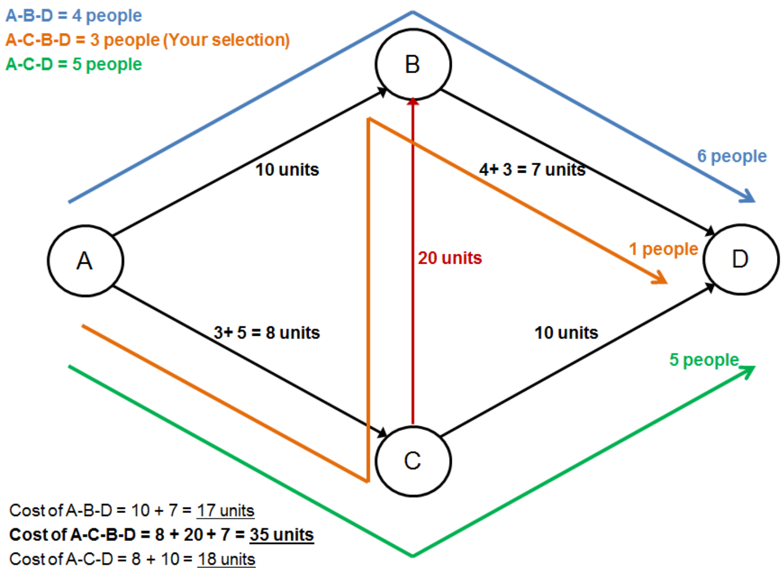

Figure 5- Costs of Routes (Incident on C-B), Round 2

As you can see the cost of travel is dependent on the **presence of the incident on C-B and the number of people selecting the path which you have selected which are both based on chance**.

Please raise your hand to indicate that you have completed reading these instructions and understood the rules of the game.

Game 1 will begin shortly.

Do NOT turn over the page until instructed to do so.

Game 2

Similar to Game 1, consider the traffic network presented in the figure below. Each driver is required to choose a route in order to travel from the starting point, denoted by *A*, to the final destination, denoted by *D*. Like before, there are three alternative routes and they are represented in the diagram as: [*A–B–D*], [*A–C–D*] and [*A-C-B-D*]. However the difference with Game 2 is that at node C you will be provided information about the state of Road C-B, analogous to a Variable Message Sign indicating the traffic conditions of the route ahead.

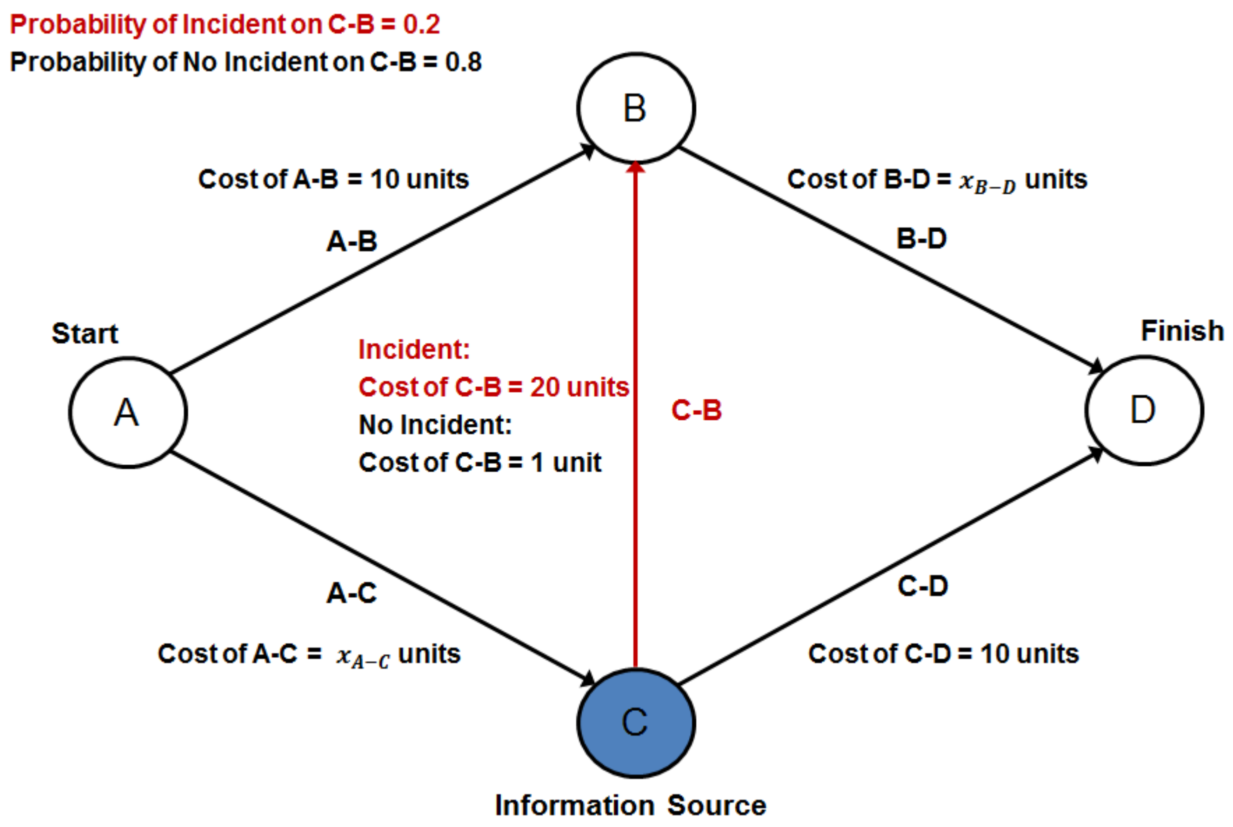

Figure 6 - Traffic Network of Game 2

The travel costs, the likelihood of an incident being present on C-B **(An incident may eventuate 20% of the time)** are identical to what was observed in Game 1. The only difference is that the presence of an incident will be revealed to you at node C if you initially choose road A-C. As with Game 1, please note that the cost charged on roads A-C and B-D of the network depend on the number of motorists choosing them.

Your activity will be to select the route which you think would be the best to travel from node A to node D.

If you travel to node C you will be provided information about the presence of an incident on C-B prior to making your route selection.

Procedure

At the beginning of each round, the computer terminal in front of you will show you the above network diagram containing the three possible routes described before; [*A–B–D*], [*A–C–D*] and [*A-C-B-D*]. The following steps are then carried out for each round

1. You will be asked to choose which of the routes you wish to travel on a road segment by road segment basis.
   a. Initially you will be asked to select between road A-B and road A-C. Once you select either option you will be asked to confirm by pressing the "Confirm" pop-up button.
   b. If you select A-B, your route is already set as there are no further choices to be made when travelling from A-D and you will be required to select B-D to complete using route [A-B-D].
   c. **If you select A-C, you will be travelling through node C where information will be provided about the state of road C-B. After being provided this information you will be asked to select between road C-B and road C-D. Once you select either option you will be asked to confirm by pressing the "Confirm" pop-up button if you are satisfied with your selection.**
   d. After receiving the information you can select the remainder of the route to reach your destination point, D.
2. After choosing a route, you will be presented with a message to wait until all other participants have made their decisions.
3. Upon the completion of the task by all participants the computer will present you with the following information;
   a. The traffic conditions of Link C-B, "No Incident on C-B" or "Incident on C-B"
   b. The route you have chosen and the payoff for the round
   c. The number of participants who chose route [*A-B-D*]
   d. The number of participants who chose route [*A-C-D*]
   e. The number of participants who chose route [*A-C-B-D*]
4. You will complete this activity for 20 rounds. At the completion of 20 rounds, the computer will inform you that Game 2 has been completed. A further message will be provided to "Please wait until the experiment continues"

All the decisions will only be made by clicking the "mouse" of the computer; there is no need to type anything whilst participating in the game.

You will receive **45 units** of income for each round. The payoff for each round will be determined by the following equation:

Payoff = (45 – Total Cost of travel) units

All 20 rounds will have the same structure.

At the end of both games (Game 1 and Game 2) you will receive payment for a randomly selected round from each of the games. You will receive **$0.25** for every unit of payoff earnt in the randomly selected rounds of each of the games.

Please raise your hand to indicate that you have completed reading these instructions and understood the rules of the game.

Game 2 will begin shortly.

Do NOT turn over the page until instructed to do so.

Thank you for completing the tasks of the experiment.

The payment calculation will commence with the central computer selecting a single random scenario from the individual choice task and a random round out of the completed 20 rounds from each game and determining your total payoff. Your final payment will be calculated as follows:

Final Payment= Attendance Fee + Individual Choice Task Payoff + $0.25*Game 1 Payoff+ $0.25*Game 2 Payoff

Your final task is to complete a short questionnaire which will be displayed on your screen shortly following this you will be paid for your participation.

You will be paid in cash for your participation within this research study.

Thank you very much for your participation today.
